# Flat vs. Expressive Storytelling: Young Children’s Learning and Retention of a Social Robot’s Narrative

**DOI:** 10.3389/fnhum.2017.00295

**Published:** 2017-06-07

**Authors:** Jacqueline M. Kory Westlund, Sooyeon Jeong, Hae W. Park, Samuel Ronfard, Aradhana Adhikari, Paul L. Harris, David DeSteno, Cynthia L. Breazeal

**Affiliations:** ^1^MIT Media Laboratory, Massachusetts Institute of TechnologyCambridge, MA, United States; ^2^Harvard Graduate School of Education, Harvard UniversityCambridge, MA, United States; ^3^Department of Psychology, Northeastern UniversityBoston, MA, United States

**Keywords:** preschool children, emotion, expressiveness, language development, peer modeling, social robotics, storytelling

## Abstract

Prior research with preschool children has established that dialogic or active book reading is an effective method for expanding young children’s vocabulary. In this exploratory study, we asked whether similar benefits are observed when a robot engages in dialogic reading with preschoolers. Given the established effectiveness of active reading, we also asked whether this effectiveness was critically dependent on the expressive characteristics of the robot. For approximately half the children, the robot’s active reading was expressive; the robot’s voice included a wide range of intonation and emotion (*Expressive*). For the remaining children, the robot read and conversed with a flat voice, which sounded similar to a classic text-to-speech engine and had little dynamic range (*Flat*). The robot’s movements were kept constant across conditions. We performed a verification study using Amazon Mechanical Turk (AMT) to confirm that the *Expressive* robot was viewed as significantly more expressive, more emotional, and less passive than the *Flat* robot. We invited 45 preschoolers with an average age of 5 years who were either English Language Learners (ELL), bilingual, or native English speakers to engage in the reading task with the robot. The robot narrated a story from a picture book, using active reading techniques and including a set of target vocabulary words in the narration. Children were post-tested on the vocabulary words and were also asked to retell the story to a puppet. A subset of 34 children performed a second story retelling 4–6 weeks later. Children reported liking and learning from the robot a similar amount in the *Expressive* and *Flat* conditions. However, as compared to children in the *Flat* condition, children in the *Expressive* condition were more concentrated and engaged as indexed by their facial expressions; they emulated the robot’s story more in their story retells; and they told longer stories during their delayed retelling. Furthermore, children who responded to the robot’s active reading questions were more likely to correctly identify the target vocabulary words in the *Expressive* condition than in the *Flat* condition. Taken together, these results suggest that children may benefit more from the expressive robot than from the flat robot.

## Introduction

Prior research with preschool children has established that storytelling and story reading can promote oral language development and story comprehension (Isbell et al., 2004; Speaker et al., 2004; Cremin et al., 2016). Participating in storytelling can increase children’s verbal fluency, listening skills and vocabulary. Book reading in particular can be an effective method for expanding young children’s vocabulary, especially when children are encouraged to actively process the story materials. For example, in an intervention study, middle class parents assigned to an experimental group were instructed to engage in “dialogic” reading with their 2-year-old, i.e., to ask more open-ended and function/attribute questions and to support the efforts of their children to answer these questions; parents in the control group were instructed to read in their usual fashion. In follow-up tests, children in the experimental group scored higher in assessments of expressive vocabulary (Whitehurst et al., 1988). Subsequent studies have replicated and extended this result (e.g., Valdez-Menchaca and Whitehurst, 1992; Hargrave and Sénéchal, 2000; Chang et al., 2012; Nuñez, 2015; Boteanu et al., 2016). Taken together, these studies indicate that dialogic book reading is an effective method for boosting children’s vocabulary. Indeed, the studies confirm that such an intervention is quite robust in its effects—it is effective for toddlers as well as preschoolers, for middle class and working class children and for typically developing as well as language-delayed children, when using print or digital storybooks.

In this exploratory study, we asked whether similar benefits could be observed when a social robot engages in dialogic story reading with preschoolers. Social robots share physical spaces with humans and leverage human means of communicating—such as speech, movement and nonverbal cues, including gaze, gestures, and facial expressions—in order to interface with us in more natural ways (Breazeal, 2004; Breazeal et al., 2008; Feil-Seifer and Mataric, 2011). Given our expectation that children would learn from the robot, we also investigated how the emotional expressiveness of the robot’s speech might modulate children’s learning.

A growing body of research suggests that social robots have potential as learning companions and tutors for young children’s early language education. For example, robots have played simple vocabulary games to help children learn new words in their own language or in a second language (Kanda et al., 2004; Movellan et al., 2009; Chang et al., 2010; Tanaka and Matsuzoe, 2012; Gordon et al., 2016; Kennedy et al., 2016). It is plausible that children’s successful vocabulary learning in these experiments depended on their relating to the robots as interactive, social beings (Kahn et al., 2013; Breazeal et al., 2016a; Kennedy et al., 2017). Social cues impact children’s willingness to engage with and learn from interlocutors (Bloom, 2000; Harris, 2007; Corriveau et al., 2009; Meltzoff et al., 2009; Sage and Baldwin, 2010). Indeed, Kuhl (2007, 2011) has argued that a lack of social interaction with a partner can impair language learning. Thus, infants learn to differentiate new phonemes presented by a live person, but do not learn this information from a video of a person, or from mere audio. Because robots are seen by children as social agents—a peer, a tutor, or a companion—they seem to be providing the necessary social presence to engage children in a language learning task. Thus, social robots, unlike educational television programs (Naigles and Mayeux, 2001), may allow children to acquire more complex language skills and not just vocabulary. However, existing studies on robots as language learning companions have generally not assessed this possibility. Nearly all of the activities performed with social robots around language learning have been simple, vocabulary-learning tasks, with limited interactivity. For example, the robot might act out new verbs (Tanaka and Matsuzoe, 2012), show flashcard-style questions on a screen (Movellan et al., 2009), or play simple give-and-take games with physical objects (Movellan et al., 2009; see also Gordon et al., 2016).

A few studies have explored other kinds of activities for language learning. For example, Chang et al. (2010) had their robot read stories aloud, ask and answer simple questions, and lead students in reciting vocabulary and sentences. However, they primarily assessed children’s engagement with the robot, rather than their language learning. One study used a story-based task in which the robot took turns telling stories with preschool and kindergarten children, for 8 weeks (Kory, 2014; Kory and Breazeal, 2014; Kory Westlund and Breazeal, 2015). In each session, the robot would tell two stories with key vocabulary words embedded, and would ask children to make up their own stories for practice. For half the children, the robot personalized the level of the stories to the child’s ability, telling more complex stories for children who had greater ability. This study found increases in vocabulary learning as well as in several metrics assessing the complexity of the stories that children produced, with greater increases when children heard appropriately leveled stories. These findings suggest that a social robot is especially likely to influence language learning if it conveys personal attunement to the child. Indeed, children were more trusting of novel information provided by a social robot whose nonverbal expressiveness was contingent on their behavior (Breazeal et al., 2016a) and showed better recall of a story when the social robot teaching them produced high immediacy gestures in response to drops in children’s attention (Szafir and Mutlu, 2012).

In the current study, we focus on a related but hitherto unexplored factor: the emotional expressiveness of the robot’s speech. Nearly every study conducted so far on the use of social robots as learning companions for young children has used a computer-generated text-to-speech voice, rather than a more natural, human voice. We know very little about the effects of a more expressive, human-like voice as compared to a less expressive, flatter or synthetic voice on children’s learning. Such expressive qualities may have an especially strong impact during storytelling activities. For example, if a potentially engaging story is read with a flat delivery, children might find it anomalous or even aversive. Using robots to study questions about expressivity is quite feasible, because we can carefully control the level of vocal expressiveness across conditions and between participants. Robots afford a level of control that it is difficult to achieve with human actors with the same consistency.

A small number of human-robot interaction (HRI) studies have investigated the effects of a robot’s voice on an interaction. However, these studies tested adults (e.g., Eyssel et al., 2012), compared different synthetic voices (e.g., Walters et al., 2008; Tamagawa et al., 2011; Sandygulova and O’Hare, 2015), or compared qualities of the same voice, such as pitch (e.g., Niculescu et al., 2013; Lubold et al., 2016), rather than varying the expressiveness of a given voice. Eyssel et al. (2012) did compare human voices to synthetic voices, but the adult participants merely watched a short video clip of the robot speaking, and did not interact with it directly. These participants perceived the robot more positively when the voice shared their gender, and anthropomorphized the robot more when the voice was human.

Some related work in speech-language pathology and education has compared children’s learning from speakers with normal human voices or voices with a vocal impairment, specifically, dysphonic voices. Children ages 8–11 years performed better on language comprehension measures after hearing passages read by a normal human voice than when the passages were read by a dysphonic voice (Morton et al., 2001; Rogerson and Dodd, 2005; Lyberg-Åhlander et al., 2015). These studies suggest that vocal impairment can be detrimental to children’s speech processing, and may force children to allocate processing to the voice signal at the expense of comprehension. However, it is unclear whether a lack of expressivity or the use of a synthetic voice would impair processing relative to a normal human voice.

Given the lack of research in this area, we compared the effect of an expressive as compared to a flat delivery by a social robot. We also focused on a more diverse population as compared to much prior work with regard to both age and language proficiency. Previous studies have tended to focus on just one population of children—either native speakers of the language, or children learning a second language—whereas we included both. In addition, few previous studies have included preschool children (Movellan et al., 2009; Tanaka and Matsuzoe, 2012; Kory, 2014); the majority of studies have targeted older children. More generally, young children comprise an age group that is typically less studied in HRI (Baxter et al., 2016).

In this study, we invited preschoolers with an average age of 5 years and considerable variation in language proficiency to engage in a dialogic reading task with a social robot. Thus, some children were English Language Learners (ELL), some were bilingual, and some were monolingual, native English speakers. All children were introduced to a robot who first engaged them in a brief conversation and then proceeded to narrate a story from a picture book using dialogic reading techniques. Two versions of the study were created; each version contained a unique set of three novel words. In post-story testing, children’s comprehension of the novel words they had heard was compared to their comprehension of the novel words embedded in the story version they had not heard. We predicted that children would display superior comprehension of the novel words that they had heard.

Given the established effectiveness of dialogic reading with young children, the robot always asked dialogic questions. We asked two related questions: first, we asked whether children would learn from a dialogic storytelling robot. Second, we asked whether its effectiveness was critically dependent on the expressiveness of the robot’s voice—how might the robot’s vocal expressivity impact children’s engagement and learning? For approximately half the children, the robot’s dialogic reading was expressive in the sense that the robot’s voice included a wide range of intonation and emotion. For the remaining children, the robot read and conversed with a flat voice, which sounded similar to a classic text-to-speech engine and had little dynamic range. To control for the many differences that computer-generated voices have from human voices (e.g., pronunciation and quality), an actress recorded both voices, and we performed a manipulation check to ensure the expressive recording was perceived to be sufficiently more emotional and expressive than the flat recording. We anticipated that children would be more attentive, show greater gain in vocabulary, and use more of the target vocabulary words themselves if the dialogic reading was delivered by the expressive as compared to the flat robot. To further assess the potentially distinct impact of the two robots, children were also invited to retell the picture-book story that the robot had narrated. More specifically, they were invited to retell the story to a puppet who had allegedly fallen asleep during the robot’s narration and was disappointed at having missed the story. Finally, a subset of children was given a second opportunity to retell the story approximately 4–6 weeks later.

## Materials and Methods

### Design

The experiment was designed to include two between-subjects conditions: Robot expressiveness (*Expressive* voice vs. *Flat* voice) and Robot redirection behaviors (*Present* vs. *Absent*). Regarding the robot’s voice, the expressive voice included a wide range of intonation and emotion, whereas the flat voice sounded similar to a classic text-to-speech engine with little dynamic range. The robot redirection behaviors were a set of re-engagement phrases that the robot could employ to redirect a distracted child’s attention back to the task at hand. However, the conditions under which the robot would use redirection behaviors did not arise—i.e., all the children were attentive and the opportunity to redirect their attention did not occur. Thus, the experiment ultimately had a two-condition, between-subjects design (*Expressive* vs. *Flat*).

### Participants

This study was carried out in accordance with the recommendations of the MIT Committee on the Use of Humans as Experimental Subjects. Children’s parents gave written informed consent prior to the start of the study and all children assented to participate, in accordance with the Declaration of Helsinki. The protocol was approved by the MIT Committee on the Use of Humans as Experimental Subjects and by the Boston Public Schools Office of Data and Accountability.

We recruited 50 children aged 4–7 (23 female, 27 male) from a Boston-area school (36 children) and the general Boston area (14 children) to participate in the study. Five children were removed from the analysis because they did not complete the study. The children in the final sample included 45 children (22 female, 23 male; 34 from the school and 11 from the general Boston area) with a mean age of 5.2 years (*SD* = 0.77). Seventeen children were ELL, eight were bilingual, 18 were native English speakers, and three were not reported.

Children were randomly assigned to conditions. There were 23 children (14 male, 9 female; 10 ELL, 6 Native English, 5 bilingual, 2 unknown) in the *Expressive* condition and 22 children (9 male, 13 female; 6 ELL, 12 Native English, 3 bilingual, 1 unknown) in the *Flat* condition. The two conditions were not perfectly balanced due to the fact that we did not obtain information about children’s language learning status until the completion of the study, and thus could not assign children evenly between conditions.

We created two versions of the story that the robot told (version A and version B); each version was identical except for the inclusion of a different set of target vocabulary words. Approximately half of the participants heard story version A (*Expressive*: 11, *Flat*: 10); the other half heard story version B (*Expressive*: 13, *Flat*: 11).

We used the Peabody Picture Vocabulary Test, 4th edition (PPVT; Dunn and Dunn, 2007), to verify that the children in the *Expressive* and *Flat* conditions did not have significantly different language abilities. The PPVT is commonly used to measure receptive language ability for standard American English. On each test item, the child is shown a page with four pictures, and is asked to point to the picture showing the target word. PPVT scores for three of the 45 children could not be computed due to missing data regarding their ages. For the remaining 42 children, there were, as expected, no significant differences between the *Expressive* and *Flat* conditions in PPVT scores, *t*_(40)_ = 0.64, *p* = 0.53. A one-way analysis of variance (ANOVA) with age as a covariate revealed that children’s PPVT scores were significantly related to their age, *F*_(3,37)_ = 5.83, *p* = 0.021, *η*^2^ = 0.114, as well as to their language status, *F*_(3,37)_ = 2.72, *p* = 0.058, *η*^2^ = 0.160. As expected, *post hoc* pairwise comparisons indicated that children who were native English speakers had higher PPVT scores (*M* = 109.4, *SD* = 18.2) than ELL children (*M* = 92.0, *SD* = 14.6), *p* = 0.004. There were no differences between the bilingual children (*M* = 103.5, *SD* = 15.7) and either the native English-speaking children or the ELL children.

### Hypotheses

The effects of the robot’s expressivity might be transient or long-term, subtle or wide-ranging. Accordingly, we used a variety of measures, including immediate assessments as well as the delayed retelling task, to explore whether the effect of the robot’s expressivity was immediate and stable and whether it impacted all measures, or selected measures only.

We tentatively expected the following results:

#### Learning

- In both conditions, children would learn the target vocabulary words presented in the story version that they heard.
- Children who learned the target words would also use them in their story retells.
- The robot’s expressivity would lead to differences in children’s long-term retention of the story. Children in the *Expressive* condition would better retain the story, and thus tell longer stories, incorporating the phrases that appeared in the initial story into their delayed retells.

#### Behavior

- In both conditions, children would typically respond to the robot’s dialogic reading questions, but children who responded more often to the dialogic reading questions would show greater learning gains.
- The *Expressive* robot would promote greater modeling by the children of the robot’s story. Children in the *Expressive* condition would produce more vocabulary and phrase mirroring.

#### Engagement

- Although most children would express liking for the robot, indirect behavioral measures would show that children were more attentive and engaged with the *Expressive* robot than with the *Flat* robot.
- The surprising moments in the story would have greater impact on children in the *Expressive* condition, because suspense and surprise in the story were strongly reflected in the robot’s voice.

### Procedure

Each child was greeted by an experimenter and led into the study area. The experimenter wore a hand puppet, a purple Toucan, which she introduced to the child: “This is my friend, Toucan.” Then the puppet spoke: “Hi, I’m Toucan!” The experimenter used the puppet to invite the child to do a standard vocabulary test, the PPVT, by saying “I love word games. Want to play a word game with me?” The experimenter then administered the PPVT.

For the children who participated in the study at their school, the PPVT was administered during an initial session. The children were brought back on a different day for the robot interaction. This second session began with the puppet asking children if they remembered it: “Remember me? I’m Toucan!” Children who participated in the lab first completed the PPVT, and were then given a 5-min break before returning to interact with the robot.

**Figure 1:**
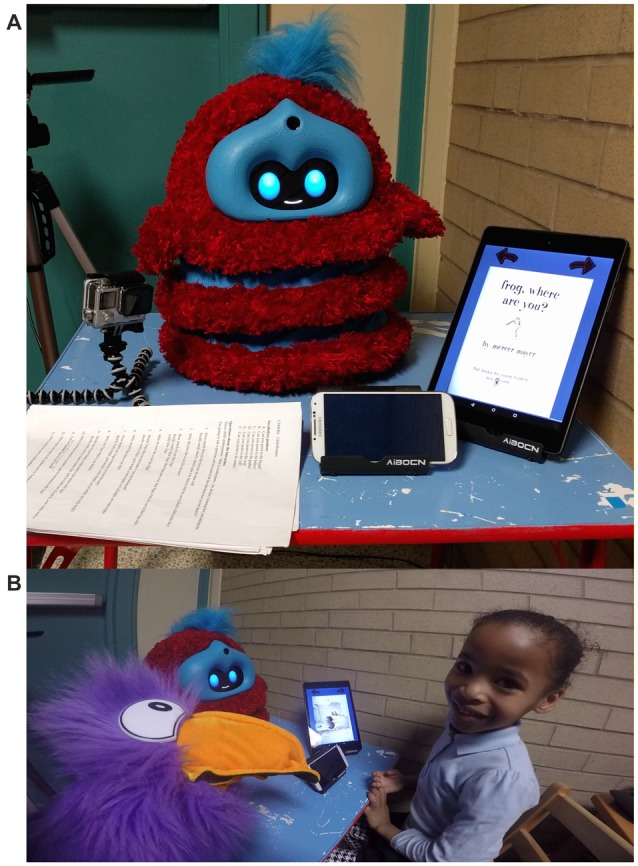
**(A)** The Tega robot sat on a table facing the child. The tablet that displayed the storybook was positioned to the right of the robot. Video cameras recorded the interaction from behind the robot, and the phone in the front used Affdex to record children’s emotional states. **(B)** A child looks up at the experimenter at the end of the robot interaction. Tega and the Toucan puppet have just said goodbye.

For the robot interaction, the experimenter led the child into the robot area. The robot sat on a low table facing a chair, in which children were directed to sit. A tablet was positioned in an upright position in a tablet stand on the robot’s right side. A smartphone sat in front of the robot; it ran software to track children’s emotional expressions (see Figure 1). The experimenter sat to the side and slightly behind the children with the puppet. The interaction began with the puppet introducing the robot, Tega: “This is my friend, Tega!” The robot introduced itself, shared personal information, and prompted children to do the same, e.g., “Hi, I’m Tega! My favorite color is blue. What is your favorite color?” and “Do you like to dance? I like to dance!”

After this brief introductory conversation, the robot asked the children if they wanted to hear a story. At this point, the puppet interjected that it was sleepy, but would try to stay awake for the story. The experimenter made the puppet yawn and fall asleep; it stayed asleep for the duration of the story. The robot then told the story which consisted of a 22-page subset of the wordless picture book “Frog, Where Are you?” by Mercer Mayer. This book has been used before in numerous studies, especially in research on speech pathology (e.g., Boudreau and Hedberg, 1999; Greenhalgh and Strong, 2001; Diehl et al., 2006; Heilmann et al., 2010).

The pages of the book were shown one at a time on the tablet screen. Each page was accompanied by 1–2 sentences of text, which the robot read in either an expressive or a flat voice depending on the condition. For every other page, the robot asked a dialogic reading comprehension question about the events in the story, e.g., “What is the frog doing?”, “Why did the boy and the dog fall?”, and “How do you think the boy feels now?” (11 questions total). The robot responded to children’s answers with encouraging, but non-committal, phrases such as “Mmhm”, “Good thought” and “You may be right”.

We embedded three target vocabulary words (all nouns) into the story. We did not test children on their knowledge of these words prior to the storytelling activity because we did not want to prime children to pay attention to these words, since that could bias our results regarding whether or not children would learn or use the words after hearing them in the context of the robot’s story. Instead, in order to assess whether children were more likely to know or use the words after hearing the robot use them in the story, two versions of the story (version A and version B) were created with different sets of target words. The two versions of the story were otherwise identical. We identified six key nouns in the original story: animal, rock, log, hole, deer and hill. Then, in each of our two story versions, we replaced three of the words with our target words, so that each story version included three target words and three original words. Version A included the target words “gopher” (original word: animal), “crag” (rock), and “lilypad” (log); version B included the words “hollow” (hole), “antlers” (deer), and “cliff” (hill). We anticipated that children would display selective learning and/or use of these words, depending on which story they heard. We looked both at children’s later receptive knowledge of the words as well as expressive or productive abilities, since children who can recognize a word may or may not be able to produce it themselves.

At the end of the story, the Toucan woke up and exclaimed, “Oh no! Did I miss the story?” This presented an opportunity for children to retell the story to the puppet, thereby providing a measure of their story recall. Children were allowed to go through the story on the tablet during their retelling. Thus, the depictions on each page could serve as a reminder during retelling.

After the story-retelling task, the experimenter administered a PPVT-style vocabulary test for the six target words used across the two versions of the story. For each word, four pictures taken from the story’s illustrations were shown to children and they were asked to point to the picture matching the target word. Finally, the experimenter asked children a set of questions regarding their perception of the robot and their enjoyment of the story. These questions were as follows:

1. How much did you like the story the robot read? Really really liked it, liked it quite a lot, liked it a little bit, sort of liked it, didn’t really like it.
2. Why did you like or not like the story?
3. How much do you like Tega? Really really liked Tega, liked Tega quite a lot, liked Tega a little bit, sort of liked Tega, didn’t really like Tega.
4. Why do you like or not like Tega?
5. Would Tega help you feel better if you were feeling sad? Really really helpful, quite helpful, a little helpful, sort of helpful, not really helpful.
6. Why would Tega help or not help?
7. How helpful was Tega in helping you learn the story? Really really helpful, quite helpful, a little helpful, sort of helpful, not really helpful.
8. Why was Tega helpful or not helpful?
9. Would one of your friends would want to read stories with Tega? Really really want to, want to quite a lot, want to a little, sort of want to, won’t really want to.
10. Why your friend would or wouldn’t want to read stories with Tega?
11. Can you describe Tega to your friend?
12. Who would you want to tell another story to: Toucan or Tega?
13. Why would you want to read another story to: Toucan or Tega?

Where appropriate, we used a Smiley-o-meter to gather responses on a 1–5 scale (Read and MacFarlane, 2006). Although Read and MacFarlane (2006) suggest that this measure is not useful with children younger than 10 years, previous research has successfully used it, or similar measures, with modest pre-training (Harris et al., 1985; Leite et al., 2014). Thus, we did a practice item before the test questions so children could learn how the measure worked. Children were also asked to explain their answers, such as “Why do you like or not like Tega?” and “Why was Tega helpful or not helpful?” Children’s parents or teachers provided demographic data regarding the children’s age and language status (ELL, bilingual, or native English speaker).

A subset of 34 children from the school sample participated in a second, follow-up session approximately 4–6 weeks later at their school. Children who participated in the lab did not have a follow-up session due to logistical reasons. During this follow-up session, we administered the PPVT a second time, then asked children to retell the story to the puppet. The puppet prompted children by saying, “I tried to tell the story to my friend last week, but I forgot most of it! Can you tell it to me again?” This allowed us to observe children’s long-term memory for the story.

Four different experimenters (three female adults and one male adult) ran the study in pairs. One experimenter interacted with the child. The other experimenter acted as the robot teleoperator and equipment manager; she could be seen by the children, but she did not interact directly with them.

### Materials

We used the Tega robot, a squash and stretch robot designed for educational activities with young children (Kory Westlund et al., 2016). The robot is shown in Figure 1. It uses an Android phone to run its control software as well as display an animated face. The face has two blue oval eyes and a white mouth, which can all morph into different shapes. This allows the face to show different facial expressions and to show appropriate visemes (i.e., mouth shapes) when speech is played back. The robot can move up and down, tilt its head sideways or forward/backward, twist to the side, and lean forward or backward. Some animations played on the robot use only the face; others incorporate both facial expressions and physical movements of the body. The robot is covered in red fur with blue stripes, giving it a whimsical, friendly appearance. The robot was referred to in a non-gendered way by the experimenters throughout the study.

A female adult recorded the robot’s speech. These utterances were shifted into a higher pitch to make them sound child-like. For the *Expressive* condition, the utterances were emotive with a larger dynamic range; the actress was instructed to speak in an expressive, human-like way. For the *Flat* condition, the actress imitated a computer-generated text-to-speech voice, keeping her intonation very flat. We did not use an actual computer-generated voice for the *Flat* voice because there would have been many differences in pronunciation and quality compared to the *Expressive* voice. Similarly, we did not use a computer-generated voice for the *Expressive* voice because no computer-generated voices can currently imitate the dynamic, expressive range that human voices are capable of.

Many of the physical actions the robot can perform are expressive. We used the same physical movements in both conditions; however, in the *Expressive* condition, some movements were accompanied by expressive sounds (such as “Mm hm!”), whereas in the *Flat* condition, these movements were either accompanied by a flat sound (“Mm hm.”) or, in cases where the sound was a short, non-linguistic expressive utterance, no sound.

We used a Google Nexus 9 8.9″ tablet to display the storybook. Touchscreen tablets have been shown to effectively engage children and social robots in a shared task (Park et al., 2014). We used custom software to display the story pages that allowed a teleoperator to control when the pages were turned; this software is open-source and available online under the MIT License at https://github.com/mitmedialab/SAR-opal-base/.

We used a Samsung Galaxy S4 android smartphone to run Affdex, which is emotion measurement software from Affectiva, Inc., Boston, MA, USA^1^. Affdex performs automatic facial coding in four steps: face and facial landmark detection, face feature extraction, facial action, and emotion expression modeling based on the EMFACS emotional facial action coding system (Ekman and Friesen, 1978; Friesen and Ekman, 1983; McDuff et al., 2016). Although no data has been published yet specifically comparing the performance of the software on adults vs. children, FACS coding is generally the same for adults and for children and has been used with children as young as 2 years (e.g., Camras et al., 2006; LoBue and Thrasher, 2015; also see Ekman and Rosenberg, 1997). Furthermore, this software has been trained and tested on tens of thousands of manually coded images of faces from around the world (McDuff et al., 2013, 2015; Senechal et al., 2015).

### Teleoperation

We used a custom teleoperation interface to control the robot and the digital storybook. Using teleoperation allowed the robot to appear autonomous to participants while removing technical barriers such as natural language understanding, because the teleoperator could be in the loop as the language parser. The teleoperator used the interface to trigger when the robot should begin its next sequence of actions (a list of speech, physical motions, and gaze) and also when the storybook should proceed to the next page. Thus, the teleoperator needed to pay attention to timing in order to trigger the robot’s next action sequence at the appropriate times relative to when the experimenter spoke (i.e., when introducing the robot to the child), or when the child responded to one of the robot’s questions. Since the teleoperator did not manage the timing of actions within each sequence, the robot’s behavior was highly consistent for all children.

The four experimenters were all trained to control the robot by an expert teleoperator; they had all controlled robots before in multiple prior studies.

### Manipulation Check

To check that the *Expressive* robot was, in fact, perceived to be more expressive than the *Flat* robot, we performed a verification study using Amazon Mechanical Turk (AMT). We recorded video of the robot performing all the speech and behavior used in the main study. We then selected samples of the robot’s speech and behavior from the introductory conversation, the beginning, middle, and end of the story, and the closing of the interaction to create a video clip that was approximately two and a half minutes in length. We created one video of the *Flat* robot and one video of the *Expressive* robot. In the two videos, we used the same speech and behavior samples such that the only difference was the expressiveness of the robot’s voice.

We recruited 40 AMT workers from the United States. Half the participants (11 male, 9 female) viewed the video of the *Flat* robot and half (13 male, 7 female) viewed the video of the *Expressive* robot. After viewing the video, participants were asked to rate their impression of the robot and report demographic information. We used the following questions, each of which was measured on a 1–5 Likert-type scale anchored with *“1: Not*___*at all”* and *“5: Extremely*___”:

1. Overall, how expressive or not expressive was the robot in the video?
2. Overall, how emotional or not emotional was the robot in the video?
3. Overall, how passive or not passive was the robot in the video?
4. How expressive or not expressive was the robot’s voice in the video?
5. How emotional or not emotional was the robot’s voice in the video?
6. How passive or not passive was the robot’s voice in the video?
7. How expressive or not expressive was the robot’s movement in the video?
8. How emotional or not emotional was the robot’s movement in the video?
9. How passive or not passive was the robot’s movement in the video?

Table 1 shows a summary of participant responses. We found that participants who watched the *Expressive* robot video rated the robot as significantly more emotional overall than participants who watched the *Flat* robot video, *t*_(39)_ = 2.39, *p* = 0.022. Participants who watched the *Expressive* robot video rated the robot’s voice as significantly more expressive, *t*_(39)_ = 4.44, *p* < 0.001; more emotional, *t*_(39)_ = 5.15, *p* < 0.001; and less passive, *t*_(39)_ = 2.96, *p* = 0.005, than participants who watched the *Flat* robot video. There were no statistically significant differences in participants’ ratings of the robot’s movement.

**Table 1:** Summary of participant responses for the *Expressive* vs. *Flat* robot verification study.

Question	Condition	Mean	Median	Mode	Range	Inter-quartile range
Overall expressive	*Flat*	3.75	4	4	1–5	1
	*Expressive*	3.60	4	4	2–5	1
Overall emotional	*Flat*	2.90	3	2	1–5	2
	*Expressive*	3.60	4	4	2–5	1
Overall passive	*Flat*	3.15	3	3	1–5	2
	*Expressive*	2.56	3	3	1–5	1
Expressive voice	*Flat*	2.65	3	1	1–5	2.25
	*Expressive*	4.05	4	4	3–5	0
Emotional voice	*Flat*	2.15	2	1	1–5	2
	*Expressive*	3.85	4	4	3–5	1
Passive voice	*Flat*	3.45	3.5	3	1–5	2
	*Expressive*	2.30	2	2	1–5	1

*The *Expressive* robot was viewed as more expressive, more emotional, and less passive than the *Flat* robot*.

The results demonstrate that the *Expressive* and *Flat* robot conditions were indeed sufficiently different from each other, with the voice of the *Expressive* robot being viewed as more expressive, more emotional, and less passive than the *Flat* robot.

### Data

We recorded video and audio data for each session using two different cameras set up on tripods behind the robot, facing the child. We recorded children’s facial expressions using Affdex, emotion measurement software from Affectiva, Inc., Boston, MA, USA. Children’s responses to the PPVT, target word vocabulary test, and interview questions were recorded on article during the experiment and later transferred to a spreadsheet.

### Data Analysis

We coded whether or not children responded to each of the questions the robot asked during the initial conversation and during the story, and if they did respond, how many words their response consisted of. We also counted the number of questions that children asked the puppet when retelling the story.

To assess how children perceived Tega as a function of their assignment to the *Expressive* and *Flat* conditions, we coded children’s responses to the open-ended question inviting them to describe Tega to a friend (i.e., “Can you describe Tega to your friend?”) for positive traits (e.g., nice, helpful, smart, fun). All children provided a response to this question. Children’s responses to the Smiley-o-meter questions were coded on a 1–5 scale.

Children’s transcribed story retells were analyzed in terms of their story length, overall word usage and target word usage, and phrase similarity compared to the robot’s original story. Automatic tools were developed such that each word was converted into its original form for comparison (stemming), words with no significant information (i.e., stopwords) were removed, and an N-gram algorithm was implemented to match phrases between the child’s and the robot’s stories. N-gram refers to a contiguous sequence of N items from a given sequence of text. In our analysis, we used *N* = 3 for matching and comparison. We chose *N* = 3 because a smaller N (e.g., *N* = 2) often retains too little information to constitute actual phrase matching, and a larger N may encompass more information than would constitute a single phrase. For example, the robot’s story included the section, “The frog jumped out of an open window. When the boy and the dog woke up the next morning, they saw that the jar was empty”. After stemming and stopword removal, this section would be converted to “frog jump open window boy dog wake next morning see jar empty”. One child retold this section of the story by saying “Frog was going to jump out the window. So whe‥. then the boy and the dog woke up, the jar was empty”. This was converted to “frog jump window boy dog wake jar empty”. The N-gram phrase matching for this segment reveals multiple phrase matches, e.g., (robot) “*frog jump* open *window”/*(child) *“frog jump window”*, and (robot) “*boy dog wake* next morning see *jar empty*”/(child) “*boy dog wake* *jar empty*”.

Children’s affect data were collected using Affdex whenever a face was detected with the front-facing camera on the Samsung Galaxy S4 device (McDuff et al., 2016). Affdex is capable of measuring 15 expressions, which are used to calculate the likelihood that the detected face is displaying each of nine different affective states. We analyzed the four affective states most relevant to our research questions: attention, concentration, surprise and engagement. *Attention* is a measure of focus based on head orientation—i.e., is the child attending to the task or not. The likelihood of *concentration* is increased by *brow furrow* and *smirk*, and decreased by *smile*. Thus, *concentration* reflects the effort and affective states associated with attending, rather than merely whether the child is looking in the correct direction or not. *Surprise* is increased by *inner brow raise*, *brow raise* and *mouth open*, and decreased by *brow furrow*. *Engagement* measures facial muscle activation reflective of the subject’s expressiveness, and is calculated as a weighted sum of the *brow raise*, *brow lower*, *nose wrinkle*, *lip corner depressor*, *chin raise*, *lip pucker*, *lip press*, *lips part*, *lip suck* and *smile*. Thus, the *Engagement* score reflects total facial muscle activation during the task. On every video frame (up to 32 frames per second), each of these affective states was scored by Affdex in the range 0 (no expression present) to 100 (expression fully present). Values in the middle (e.g., 43 or 59) indicate that the expression is somewhat present; these values are relative and Affdex does not indicate what the exact difference is between each score. See Senechal et al. (2015) for more detail regarding the algorithms uses for classification.

For the story retelling, the audio quality of 40 out of 45 participants was sufficiently good enough for transcription (22 female, 18 male; age *M* = 5.2, *SD* = 0.76; 14 ELL, 7 bilingual, 16 native English, 3 unknown). There were 21 children (10 female, 11 male; age *M* = 5.3, *SD* = 0.80; 9 ELL, 4 bilingual, 6 native English, 2 unknown) in the *Expressive* condition and 19 children (12 female, 7 male; age *M* = 5.1, *SD* = 0.71; 5 ELL, 3 bilingual, 10 native English, 1 unknown) in the *Flat* condition. Half of the participants had heard story version A (*Expressive*: 10, *Flat*: 10); the other half had heard story version B (*Expressive*: 11, *Flat*: 9).

To perform analyses across the two sessions, immediate and delayed retell pairs from 29 children were used (14 female, 15 male; age *M* = 5.2, *SD* = 0.68; 14 ELL, 3 bilingual, 12 native English). There were 15 children (6 female, 9 male; age *M* = 5.3, *SD* = 0.70; 9 ELL, 2 bilingual, 4 native English) from the *Expressive* condition and 14 children (8 female, 6 male; age *M* = 5.1, *SD* = 0.66; 5 ELL, 1 bilingual, 8 native English) from the *Flat* condition. Half of the participants heard story version A (*Expressive*: 8, *Flat*: 8); the other half heard story version B (*Expressive*: 7, *Flat*: 6).

In the following analyses, we ran Shapiro-Wilk (S-W) tests to check for normality and Levene’s test to check for equal variance, where applicable. Levene’s null hypothesis was rejected for all data in our dataset (*p* > 0.05) and constant variance was assumed across conditions and sessions. Parametric (paired/unpaired *t-test*) and non-parametric (Wilcoxon signed-rank and Mann-Whitney’s U) tests were used based on the S-W result.

## Results

We present our results in three parts, with each part addressing one of our three main hypotheses: (1) *Learning*: our primary question was whether children would learn from a robot that led a dialogic storytelling activity, and specifically whether the expressiveness of the robot’s voice would impact children’s learning; (2) *Behavio*r: we asked whether children would learn more if they responded to the dialogic reading questions, and whether the robot’s expressiveness would produce greater lexical and phrase modeling; and (3) *Engagement*: we asked whether the robot’s expressiveness would lead to greater attention or engagement. Finally, we also examined whether children’s learning was impacted by their language status.

### Learning

#### Target Vocabulary Word Identification

Overall, children correctly identified a mean of 4.0 of the six target vocabulary words (*SD* = 1.38). A 2 × 2 × 2 mixed ANOVA with condition (*Expressive* vs. *Flat*), the story children heard (version A vs. version B), and the words correctly identified (number of version A words correct vs. number of version B words correct, where children were asked to identify both sets of words), with age as a covariate, revealed a trend toward age affecting how many words children identified correctly, *F*_(1,81)_ = 3.40, *p* = 0.069, *η*^2^ = 0.045. *Post hoc* pairwise comparisons showed that older children identified more target words correctly, with 4-year-olds identifying fewer words than 5-year-olds (*p* = 0.016), 6-year-olds (*p* = 0.016), and the 7-year-old (*p* = 0.077; Table 2). There was no difference between the total number of target vocabulary words that children identified correctly in the *Expressive* (*M* = 3.8 correct of 6, *SD* = 1.48) vs. *Flat* (*M* = 4.23, *SD* = 1.27) conditions.

**Table 2:** Older children to correctly identified more of the target vocabulary words.

Age	Number of children	Mean target words correct *(SD)*
4 years	9	3.22 *(1.30)*
5 years	21	4.14 *(1.28)*
6 years	14	4.21 *(1.53)*
7 years	1	5.00 *(N/A)*

**Figure 2:**
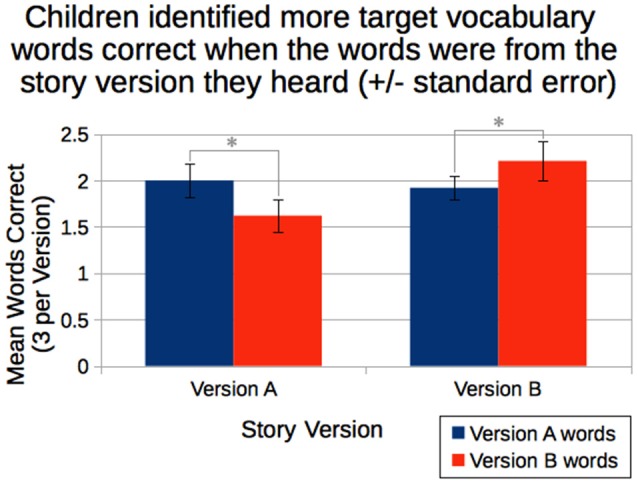
Children who heard story version A correctly identified more version A words than version B words, whereas children who heard story version B correctly identified more version B words than version A words. *Statistically significant at *p* < 0.05.

We also found the expected interaction between story version heard and number of words correctly identified from each version (Figure 2). Children who heard story version A were likely to correctly identify more version A words (*M* = 2.00 correct of 3, *SD* = 0.853) than version B words (*M* = 1.62, *SD* = 0.813), whereas children who heard story version B were more likely to correctly identify more version B words (*M* = 2.21 correct of 3, *SD* = 1.03) than version A words (*M* = 1.92, *SD* = 0.626), *F*_(1,81)_ = 4.21, *p* = 0.043.

In summary, performance in the vocabulary test improved with age. Nevertheless, there was evidence of learning from the story in that children performed better on those items they had encountered in the story version they heard.

#### Target Word Use

**Figure 3:**
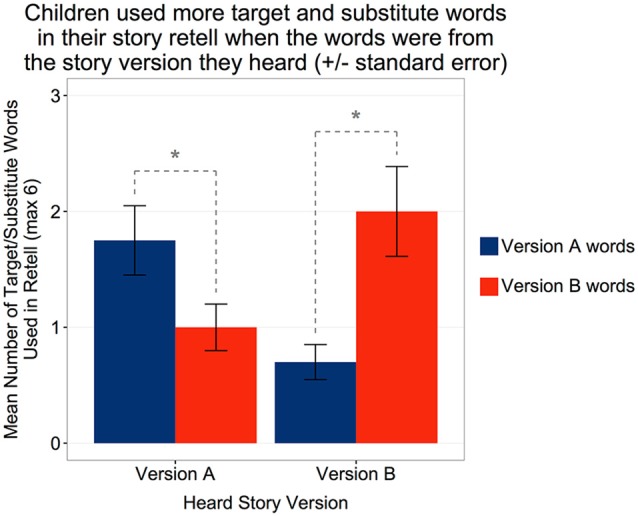
Children who heard story version A used more version A target and original words than version B words, whereas children who heard story version B used more version B target and original words than version A words in their immediate story retell. *Statistically significant at *p* < 0.05.

First, because the two story versions (A and B) differed both in terms of the target words included and the original words (i.e., the lower level words that we replaced with the target words), we analyzed how often children used either type of word. This was to provide context in terms of children’s overall word reuse rates after hearing the words in the robot’s story. Thus, among 40 children, 35 children either used the target words or the original words in their story retelling (*M* = 2.15 out of 6, *SD* = 1.48). As in the target word identification, we also found significant differences in children’s word usage behavior based on the story version they heard. A Wilcoxon signed-rank test revealed that children who heard story version A were more likely to use version A words in their story retelling (*M* = 1.75, *SD* = 1.37) than version B words (*M* = 1.00, *SD* = 0.920); *W* = 12, *Z* = −2.34, *p* = 0.019, *r* = 0.52, whereas children who heard story version B were more likely to use version B words (*M* = 2.00, *SD* = 1.69) than version A words (*M* = 0.700, *SD* = 0.660); *W* = 12.5, *Z* = −2.87, *p* = 0.004, *r* = 0.64 (Figure 3).

Then, to analyze children’s learning of new words from the robot, we focused on children’s reuse of the target words. There was no significant difference in overall target word usage between the *Flat* and *Expressive* conditions. In the immediate retell, children used a mean of 0.45 target words (out of 3), *SD* = 0.69. However, out of the 17 children who used at least one of the target words in their retell (*Expressive*: 10 children, *Flat*: 7), children in the *Expressive* condition used significantly more target words (*M* = 1.6, *SD* = 0.70) than children in the *Flat* condition (*M* = 1.00, *SD* = 0.00), *t*_(15)_ = 2.248, *p* = 0.040.

**Figure 4:**
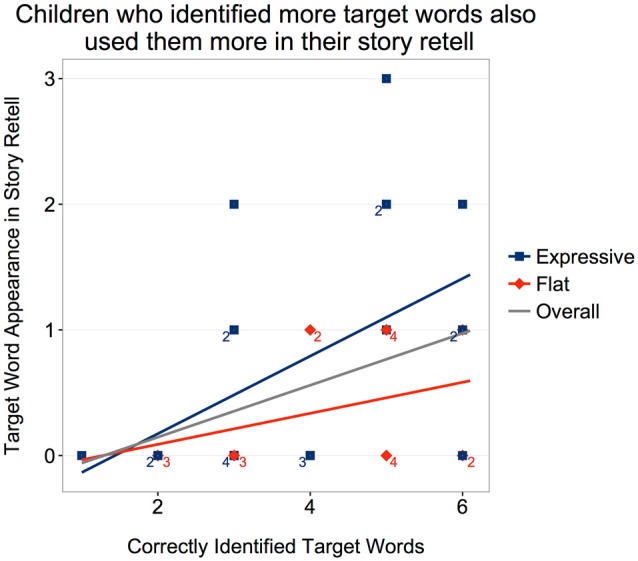
Children who correctly identified more target words also used them more in their story retell. The trend was primarily driven by the Expressive condition.

A trend toward older children using more target words than younger children was also observed; age 4 (*M* = 0.14, *SD* = 0.38), age 5 (*M* = 0.58, *SD* = 0.77), age 6 (*M* = 0.62, *SD* = 0.65), age 7 (*M* = 3.0, *SD* = 0.00); Kendall’s rank correlation *τ*_(38)_ = 0.274, *p* = 0.059. In the delayed retell, time was significant (*M* = 0.21, *SD* = 0.49; *W* = 10, *Z* = −2.77, *p* = 0.05). The correlation between the number of target words that children used in the immediate retell and their score on the target-word test was significant, *τ*_(38)_ = 0.348, *p* = 0.011. This trend was significant in the *Expressive* condition (*τ*_(19)_ = 0.406, *p* = 0.031), but not in the *Flat* condition (*τ*_(17)_ = 0.246, *p* = 0.251; Figure 4).

In summary, children tended to use more of the target words encountered in the story version they heard, and older children tended to use more of the target words.

#### Story Length

The length of the story told by the robot was 365 words. In the immediate retell, the mean length of children’s stories was 200.7 words (*SD* = 80.8). No statistically significant difference in story length was observed between the two conditions (*Expressive*: *M* = 191.8 words, *SD* = 82.5, *Flat*: *M* = 210.6, *SD* = 79.9), *t*_(38)_ = −0.73, *p* = 0.47. Story length also did not vary with age, Pearson’s *r*_(7)_ = 0.06, *p* = 0.71.

A 2 × 2 mixed ANOVA with time (within: Immediate vs. Delayed) and condition (between: *Expressive* vs. *Flat*) for the subset of children who produced both immediate and delayed retells revealed significant main effects of time, *F*_(1,27)_ = 17.9, *p* < 0.001, *η*^2^ = 0.398, as well as a significant interaction between time and condition, *F*_(1,27)_ = 15.0, *p* < 0.001, *η*^2^ = 0.357. In the delayed retell, the overall length of children’s story decreased to *M* = 147.9 (*SD* = 58.3; *t*_(13)_ = 5.35, *p* < 0.001). Children in the *Flat* condition showed a significant decrease (Immediate: *M* = 210.9, *SD* = 85.4, Delayed: *M* = 125.4, *SD* = 57.2), while in the *Expressive* condition, the decrease was not statistically significant (Immediate: *M* = 173.3, *SD* = 79.33, Delayed: *M* = 168.9, *SD* = 52.8; *t*_(14)_ = 0.33, *p* = 0.75). Furthermore, the length of stories in the two conditions were significantly different at the delayed retelling (*Expressive*: *M* = 168.9, *SD* = 52.8, *Flat*: *M* = 125.4, *SD* = 57.2), *t*_(27)_ = 2.13, *p* = 0.043.

**Figure 5:**
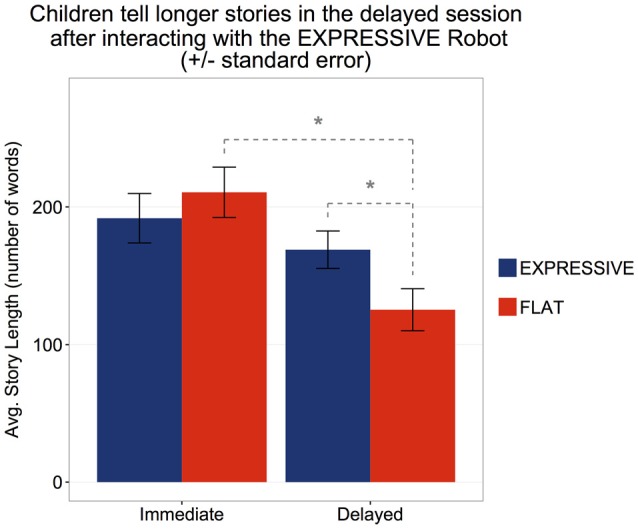
Children’s story-retell length significantly reduced after 1–2 months in the Flat condition, but not in the Expressive condition. *Statistically significant at *p* < 0.05.

Thus, children in the *Flat* condition told shorter stories at the delayed retell as compared to the immediate retell whereas no such reduction was seen among children in the *Expressive* condition. Their stories were just as lengthy after 1–2 months (Figure 5). To further understand the impact of expressivity on retelling, we analyzed children’s phrase production as reported in the following section.

### Behavior

#### Responses to the Robot’s Dialogic Questions

Forty-two children had data regarding their responses to the robot-posed dialogic reading questions. Thirty-five (83.3%) responded to at least some of the questions; 23 (54%) responded to all 11 questions; seven (16.7%) responded to none. There was no significant difference between the number of questions responded to by children in the *Expressive* and *Flat* conditions.

**Figure 6:**
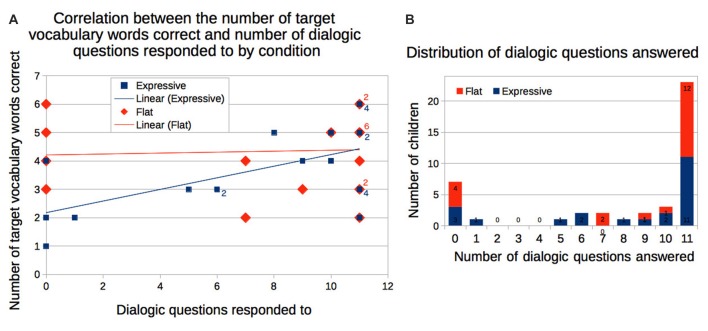
**(A)** Children who responded to the robot’s dialogic questions were also more likely to correctly identify more of the target vocabulary words. The correlation was primarily driven by children in the Expressive condition. **(B)** The majority of children responded to most or all of the robot’s dialogic questions.

A simple linear regression model revealed that children who had responded to the robot’s dialogic questions were likely to correctly identify more of the target vocabulary words, *F*_(1,38)_ = 5.84, *p* = 0.021, *η*^2^ = 0.118. The interaction between the condition and the number of questions responded showed a trend, *F*_(1,38)_ = 4.094, *p* = 0.0501, *η*^2^ = 0.083, such that question answering in the *Expressive* condition was related to correct identification of target words, while question answering was not related to correct identification of words in the *Flat* condition. The correlation was driven primarily by the *Expressive* condition, *r*_(20)_ = 0.619, *p* = 0.002, i.e., children in the *Expressive* condition who answered the robot’s questions were more likely to identify more of the target words; there was no significant correlation for the *Flat* condition, *r*_(18)_ = 0.134, *p* = 0.57 (Figure 6). Thus, answering the dialogic questions was linked to better vocabulary learning, but this link was only found in the *Expressive* condition.

**Figure 7:**
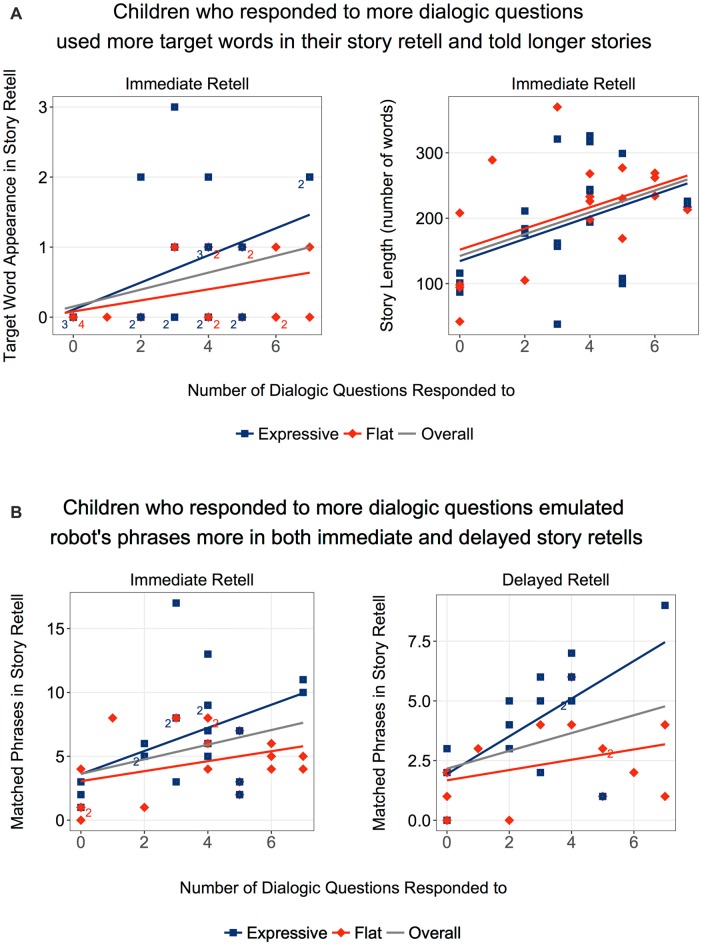
**(A)** Children who responded to the robot’s dialogic questions were more likely to use the target words in their retells and tell longer stories. **(B)** These children were also more likely to emulate robot’s story in terms of phrase similarity, in both immediate and delayed retell. The trend was primarily driven by the *Expressive* condition.

Children who answered more dialogic questions also used significantly more target words in the immediate story retell as indicated by a Spearman’s rank-order correlation *r_s_*_(38)_ = 0.352, *p* = 0.026. These children also told longer stories, *r*_s_(38) = 0.447, *p* = 0.003 (Figure 7A). They displayed greater emulation of the robot in terms of phrase usage, *r*_s_(38) = 0.320, *p* = 0.044, but again this was driven primarily by the *Expressive* condition, *r*_s_(19) = 0.437, *p* = 0.048, and not by the *Flat* condition, *r*_s_(17) = 0.274, *p* = 0.257. Children in the *Expressive* condition also showed significant correlation to phrase usage in the delayed retell, *r*_s_(13) = 0.554, *p* = 0.032 (Figure 7B).

From the above observations, we can conclude that children were, in general, actively engaged in the robot’s storytelling. When children were more engaged, as indexed by how often they responded to the robot’s questions, their vocabulary learning was greater, and they were more likely to emulate the robot. However, these links between engagement and learning were evident in the *Expressive* rather than the *Flat* condition.

#### Emulating the Robot’s Story

An analysis of children’s overall word usage reveals their word-level mirroring of the robot’s story. In total, the robot used 96 unique words after stopword removal and the calculation of non-overlapping words. In the immediate retell, children used a mean of 58.7 words (*SD* = 12.4) emulating the robot. There was no significant difference between conditions. In the delayed retell, however, children in the *Expressive* condition used more words emulating the robot than children in the *Flat* condition (*Expressive*: *M* = 48.6, *SD* = 13.5, *Flat*: *M* = 38.7, *SD* = 8.62; *t*_(27)_ = 2.33, *p* = 0.028).

**Figure 8:**
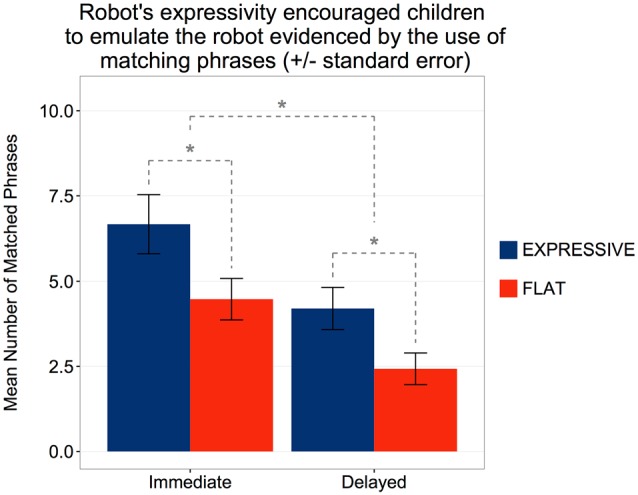
Children in the Expressive condition showed stronger emulation of the robot’s story in terms of phrase similarity, in both immediate and delayed retell. *Statistically significant at *p* < 0.05.

We also analyzed the phrase-level similarity between the robot’s story and the children’s stories. In the immediate retell, a mean of 5.63 phrases (*SD* = 3.55) were matched. A statistically significant difference was observed between conditions (*Expressive*: *M* = 6.67, *SD* = 3.98, *Flat*: *M* = 4.47, *SD* = 2.65; *t*_(38)_ = 2.03, *p* = 0.049) with robot’s expressivity increasing children’s phrase-level similarity. In the delayed retell, the overall usage of matched phrases decreased (*M* = 3.34, *SD* = 2.26), *t*_(28)_ = 5.87, *p* < 0.001. However, a Mann-Whitney U test showed that participants in the *Expressive* condition (*M* = 4.20, *SD* = 2.40) continued to use more similar phrases than participants in the *Flat* condition (*M* = 2.42, *SD* = 1.74), *Z* = 2.07, *p* = 0.039, *r* = 0.38 (Figure 8). Thus, at both retellings, children were more likely to echo the expressive than the flat robot in terms of their phrasing.

**Figure 9:**
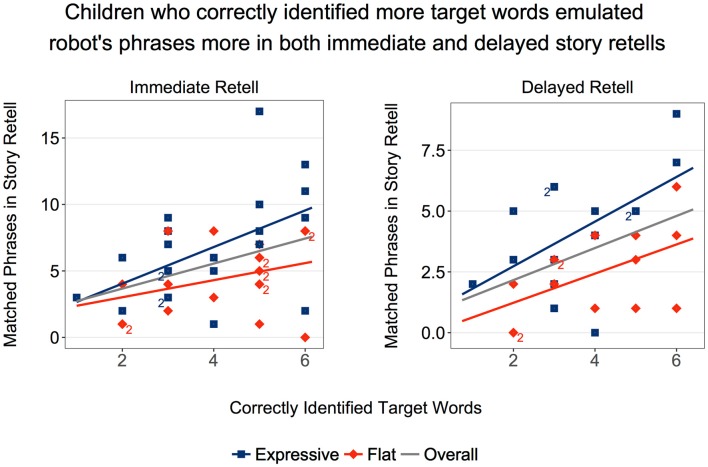
Children who correctly identified more target words were also more likely to emulate the robot’s story in terms of phrase similarity, in both immediate and delayed retell. These trends were primarily driven by the *Expressive* condition.

The overall correlation between children’s score on the target-word vocabulary test and the number of matched phrases they used in the retell was significant both for the immediate retell, *r_s_*_(38)_ = 0.375, *p* = 0.017; and for the delayed retell, *r*_s_(27) = 0.397, *p* = 0.033 (Figure 9). However, further analysis showed that this link was significant in the *Expressive* condition (Immediate: *r*_s_(19) = 0.497, *p* = 0.022; Delayed: *r*_s_(13) = 0.482, *p* = 0.031), but not in the *Flat* condition (Immediate: *r*_s_(19) = 0.317, *p* = 0.186; Delayed: *r*_s_(12) = 0.519, *p* = 0.067).

In summary, children were more likely to use similar words and phrases as the robot in the *Expressive* than in the *Flat* condition during both retellings. Furthermore, given that scores on the target-word vocabulary test were not significantly different between the two conditions, the correlation results suggest that the robot’s expressivity did not impact initial encoding, but did encourage children to emulate the robot in their subsequent retelling of the story.

### Engagement

#### Interview Questions

We found no difference between conditions in children’s responses to the interview questions. Children reported that they liked the story (*Median* = 5, *Mode* = 5, *Range* = 1–5, *Inter-Quartile Range (IQR)* = 1) and that they liked Tega (*Median* = 5, *Mode* = 5, *Range* = 3–5, *IQR* = 0). For example, one child said said he liked the story because “in the end they found a new pet frog”. Children’s reasons for liking Tega included physical characteristics, such as “furry”, “cute”, and “red”, as well as personality traits including “kind” and “nice”.

Children thought Tega could help them feel better (*Median* = 5, *Mode* = 5, *Range* = 1–5, *IQR* = 0), saying, for example, that “he’s cute, funny, and makes me smile”, and “would give a big hug”. They thought Tega helped them learn the story (*Median* = 5, *Mode* = 5, *Range* = 2–5, *IQR* = 0). One child reported Tega was helpful because “the story was a little bit long”, while another said “because he asked me what happened in the story”. Another child also noted the questions, saying “stopped to ask questions and talked slowly so I could understand”. Children thought their friends would like reading with Tega (*Median* = 5, *Mode* = 5, *Range* = 1–5, *IQR* = 0), because “he’s a nice robot and will be nice to them”, and “Tega’s got a lot of good stories, and is good at telling them”.

When asked if they would prefer to play again with Tega or with the Toucan puppet, 26 children picked Tega, 11 picked Toucan, and 8 either said “both”, “not sure”, or did not respond. They justified picking Toucan with reasons such as “Toucan didn’t hear the story”, “because he fell asleep and is super, super soft”, and “because she’s very sleepy and never listens”. They justified picking Tega with various reasons including “because Tega can listen and Toucan is just a puppet”, “because she read the story to me”, “because he’s fun”, and “I like her”. Thus, we see that children felt the desire to be fair in making sure Toucan got a chance to hear the story, and a desire to reciprocate Tega’s sharing of a story with them, as well as expressing general liking for the robot.

When asked to describe Tega to a friend, 44% of children described the robot using positive traits (e.g., nice, helpful, smart, fun) in the *Expressive* condition and 48% in the *Flat* condition, *ns*. For example, one child said, “he told me about antlers. Tega is very helpful”, while another reported “that he read me a story and will be a nice robot to them”. In sum, the expressiveness of the robot did not influence how children described the robot to a peer. Many of the other 56% of children in the *Expressive* condition and the 52% of children in the *Flat* condition focused on the robot’s physical characteristics, for example, “red and blue, stripes, big eyes, tuft of blue hair, phone for face, fuzzy, cute smile”. One child said Tega “looks like a rock star”.

#### Children’s Expressivity

We analyzed affect data for 36 children (19 in the *Expressive* condition and 17 in the *Flat* condition). For the remaining nine children, no affect data were collected either because the children’s faces were not detected by the system, or because of other system failures.

As described earlier, we focused our analysis on the four affective states most relevant to our research questions: concentration, engagement, surprise and attention. All other affective states were measured by Affdex very rarely (less than 5% of the time). We found that overall, children maintained attention throughout most of the session, were engaged by the robot, showed some concentration, and displayed surprise during the story (Table 3).

**Table 3:** Analysis of four facial expressions during the interaction by condition.

Expression	Overall mean (SD)	Expressive mean (SD)	Flat mean (SD)
Concentration	11.7 (7.63)	14.1 (8.33)	8.93 (5.83)
Engagement	20.8 (12.1)	24.5 (12.6)	16.6 (10.3)
Surprise	6.71 (4.57)	8.28 (4.99)	4.95 (3.39)
Attention	82.6 (7.45)	82.4 (7.47)	83.0 (7.63)

*Values can range from 0 (no expression present) to 100 (expression fully present)*.

**Figure 10:**
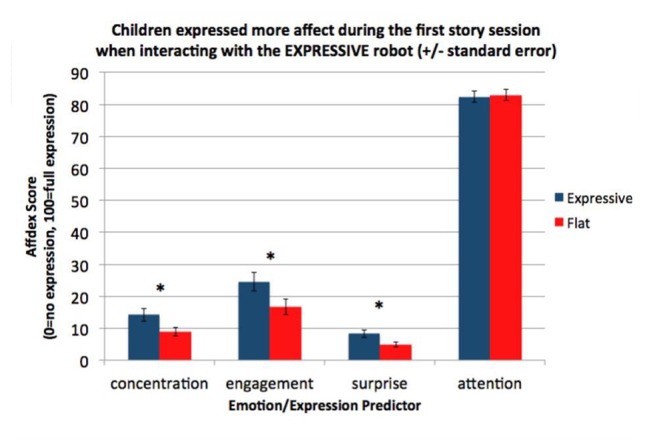
Children in the Expressive condition showed more concentration, engagement and surprise during the session than children in the Flat condition. Attention levels were not statistically different between the two conditions. *Statistically significant at *p* < 0.05.

To evaluate whether the robot’s vocal expressiveness influenced children’s facial expressiveness, we examined the mean levels of the four affective states across the entire session by condition. We conducted a one-way ANCOVA with condition (*Expressive* vs. *Flat*) for each Affdex score, with age as a covariate. The analysis revealed that children in the *Expressive* condition showed significantly higher mean levels of concentration, *F*_(1,32)_ = 4.77, *p* = 0.036, *η*^2^ = 0.127; engagement, *F*_(1,32)_ = 4.15, *p* = 0.049, *η*^2^ = 0.112; and surprise, *F*_(1,32)_ = 5.21, *p* = 0.029, *η*^2^ = 0.13, than children in the *Flat* condition, but that children’s attention was not significantly different, *F*_(1,32)_ = 0.111, *p* = 0.741. Furthermore, these differences were not affected by children’s age (Table 3, Figure 10). The lack of difference in children’s attention demonstrated that the differences in the concentration, engagement and surprise levels across the two conditions were not a result of children paying less attention to the Flat robot’s story.

**Figure 11:**
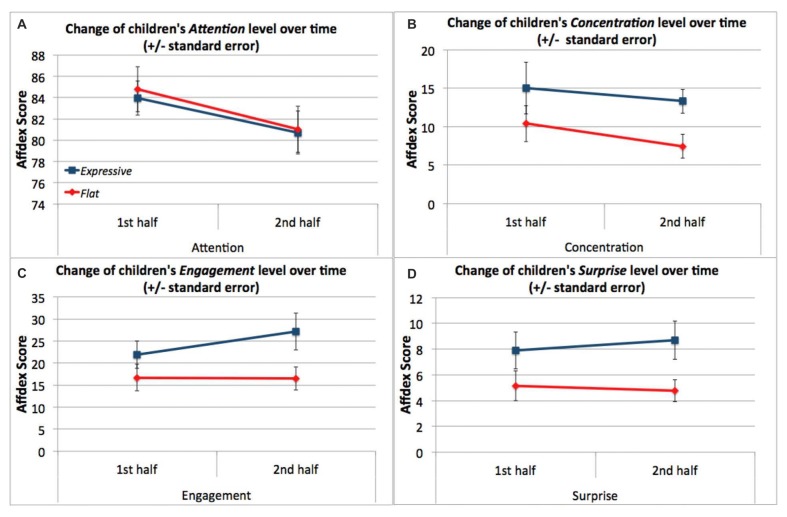
**(A)** Children’s level of attention decreased in the course of the session but showed no difference between conditions. **(B)** The concentration level of children in the Expressive condition was consistently higher than that of children in the Flat condition. **(C)** The engagement level of children in the Expressive condition was consistently higher than that of children in the Flat condition. **(D)** The surprise level of children in the Expressive condition was consistently higher than that of children in the Flat condition.

Next, we asked whether children’s affect changed during the session. We split the affect data into two halves—the first half of the session and the second half of the session—using the data timestamps to determine the session halfway point We ran a 2 × 2 mixed design ANOVA with time (within: first half vs. second half) × condition (between: *Expressive* vs. *Flat*) for each of the affect scores. These analyses revealed main effects of condition on children’s concentration scores, *F*_(1,34)_ = 4.71, *p* = 0.037, *η*^2^ = 0.067; engagement scores, *F*_(1,34)_ = 4.16, *p* = 0.049, *η*^2^ = 0.075; and surprise scores, *F*_(1,34)_ = 5.36, *p* = 0.027, *η*^2^ = 0.090. In all three cases, children displayed greater affect in the *Expressive* condition than the *Flat* condition (see Figures 11B–D). There were no main effects of time or any significant interactions for these affect measures. However, we did see a main effect of time for children’s attention scores, *F*_(1,34)_ = 7.84, *p* = 0.008, *η*^2^ = 0.044. In both conditions, children’s attention scores declined over time (Figure 11A).

In summary, although all children were less attentive over time, they showed more facial expressiveness throughout the whole session with the expressive robot than with the flat robot.

### Language Status

We completed our analyses by checking whether the results were stronger or weaker based on children’s language status (i.e., native English speakers, ELL, or bilingual). The differences were modest and are reported here.

**Figure 12:**
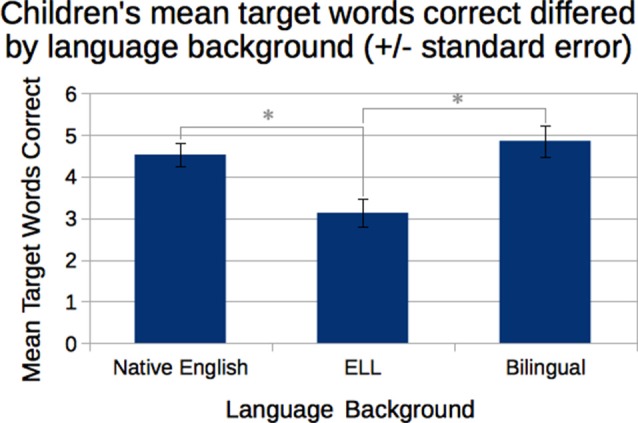
Children who were native English speakers or bilingual correctly identified more of the target vocabulary words than did English Language Learners (ELL) children. *Statistically significant at *p* < 0.05.

First, with regards to learning new vocabulary, a one-way ANOVA with age as a covariate revealed that children’s language status affected how many target vocabulary words they identified correctly, *F*_(3,37)_ = 4.10, *p* = 0.012, *η*^2^ = 0.230, but vocabulary learning was not affected by age (Figure 12). *Post hoc* pairwise comparisons showed that children who were native English speakers correctly identified more words (*M* = 4.53 correct, *SD* = 1.23) than ELL children (*M* = 3.13 correct, *SD* = 1.30), *p* = 0.002. Bilingual speakers also identified more words correctly (*M* = 4.86, *SD* = 1.07) than ELL children, *p* = 0.005, but were not significantly different from the native English speakers.

Second, native English speakers used more target words (*M* = 0.94, *SD* = 0.93) than ELL students (*M* = 0.14, *SD* = 0.36) in the immediate retell, *t*_(20)_ = −3.16, *p* = 0.005. Bilingual students were in-between (*M* = 0.29, *SD* = 0.49). This trend was primarily driven by the *Expressive* condition, *F*_(2,34)_ = 5.458, *p* = 0.009, rather than the *Flat* condition. *Post hoc* pairwise comparison within the *Expressive* condition showed that native speakers used more target words than bilingual speakers, *t*_(8)_ = 3.00, *p* = 0.017, and more than ELL speakers, *t*_(13)_ = 7.45, *p* < 0.001. Bilingual speakers also used more target words than the ELL group in the immediate retell, *t*_(11)_ = 2.75, *p* = 0.019.

Lastly, native English speakers showed stronger phrase mirroring behavior (*M* = 6.56, *SD* = 4.49) than ELL students (*M* = 4.43, *SD* = 2.38) in the *Expressive* condition in the immediate retell, *t*_(13)_ = 3.41, *p* = 0.005. The robot’s expressivity had a significant effect on native English speakers’ usage of similar phrases in both the immediate retell (*Expressive* *M* = 10.17, *SD* = 4.40, *Flat* *M* = 4.40, *SD* = 2.99), *t*_(14)_ = 3.139, *p* = 0.007; and in the delayed retell (*Expressive* *M* = 5.50, *SD* = 2.52, *Flat* *M* = 2.38, *SD* = 1.30), *t*_(10)_ = 2.904, *p* = 0.016. Though not significant, ELL children trended toward also using more similar phrases when they heard the story from the *Expressive* robot in both the immediate retell (*Expressive* *M* = 4.55, *SD* = 1.94, *Flat* *M* = 4.20, *SD* = 3.27) and the delayed retell (*Expressive* *M* = 3.78, *SD* = 1.86, *Flat* *M* = 2.20, *SD* = 2.49).

In summary, as might be expected, native English speakers performed better on the vocabulary test, used more target words, and showed more phrase matching than either ELL or bilingual children.

## Discussion

We asked whether children would learn from a dialogic, storytelling robot and whether the robot’s effectiveness as a narrator and teacher would vary with the expressiveness of the robot’s voice. We hypothesized that a more expressive voice would lead to greater engagement and greater learning. Below, we review the main findings pertinent to each of these questions and then turn to their implications.

Whether the robot spoke with a flat or expressive voice, children were highly attentive in listening to the robot—as indexed by their head orientation—when it was recounting the picture book story. Moreover, irrespective of the robot’s voice, children were able to acquire new vocabulary items embedded in the story. Although some children may have already known some of the target words, as indicated by their above-zero recognition of the target words from the story version they did *not* hear, the interaction between story version heard and scores on each set of words (shown in Figure 2) shows that genuine learning did occur. Children could also retell the story (with the help of the picture book) both immediately afterwards and some weeks later. At their initial retelling, children typically produced a story about half as long as the one they had heard, sometimes including a newly acquired vocabulary item. Finally, when they were invited to provide both an explicit evaluation and a free-form description, children were equally positive about the robot whether they had listened to the flat or the expressive robot.

Despite this equivalence with respect to attentiveness, encoding and evaluation, there were several indications that children’s mode of listening was different for the two robots. First, as they listened to the expressive rather than the flat robot, children’s facial expressions betrayed more concentration (i.e., more brow furrowing and less smiling), more engagement (i.e., greater overall muscle activation) and more surprise (i.e., more brow raising with open mouth). Thus, children were not only attentive to what the robot was saying, they also displayed signs of greater emotional engagement.

Furthermore, inclusion of the newly acquired vocabulary items in the initial retelling was more frequent among children who listened to the expressive rather than the flat robot. Note that children’s score on the target-word test was not significantly different between the two conditions, suggesting that children who correctly identified the target words in the *Expressive* condition tended to also use them in their story retell whereas children who correctly identified the target words in the *Flat* condition were less likely to use them in their story recall. Thus, although children were able to acquire new vocabulary from either robot (*receptive* vocabulary knowledge), they were more likely to subsequently use that vocabulary in their stories if the expressive robot had been the narrator (i.e., *productive* vocabulary knowledge). This pattern of findings implies that children could encode and retain new input from either robot, but they were more likely to engage with the expressive robot during the narration and more likely to emulate the expressive robot’s narrative vocabulary in their own recounting. That is, interacting with the expressive robot led to greater *behavioral* outcomes—producing new words rather than merely identifying them.

Further signs of the differential impact of the two robots were found at the delayed retelling. Whereas there was a considerable decline in story length among children who had heard the flat robot, there was no such decline among children who had heard the expressive robot. Again, we cannot ascribe this difference to differences in encoding. Children in each condition had told equally long stories on their initial retelling. A more plausible interpretation is that children who had heard the expressive robot were more inclined to emulate its narrative than children who had heard the flat robot. More detailed support for this interpretation emerged in children’s story phrasing. At both retellings, children were more likely to echo the expressive rather than the flat robot in terms of using parallel phrases. This may also indicate that children were engaging with the expressive robot as a more socially dynamic agent, since past research has shown that children are more likely to use particular syntactic forms when primed by an adult (e.g., Huttenlocher et al., 2004). In addition, recent work by Kennedy et al. (2017) showed that a robot that used more nonverbal immediacy behaviors (e.g., gestures, gaze, vocal prosody, facial expressions, proximity and body orientation, touch) led to greater short story recall by children. The difference in the expressive vs. flat robot’s vocal qualities (e.g., intonation and prosody) could have led to a difference in the perceived nonverbal immediacy of the robot, which may have led to the differences in children’s engagement with the robot as a socially dynamic agent.

Both the expressive and the flat robot asked dialogic questions about the story as they narrated it. The more often children answered these dialogic questions the more vocabulary items they learned. Here too, however, the robot’s voice made a difference. The link between question answering and vocabulary acquisition was only significant for the *Expressive* condition. Children who answered more dialogic questions also displayed greater fidelity to the robot’s story in terms of phrase usage when they retold it, but again this link was only significant for the *Expressive* condition. Thus, answering more of the robot’s questions was associated with the acquisition of more vocabulary and greater phrase emulation but only for the expressive robot. Finally children’s score on the target-word vocabulary test correlated with the number of matched phrases they used at both retellings. However, this correlation emerged only for children in the expressive condition, again consistent with the idea that robot expressivity enhanced emulation but not initial encoding.

In sum, we obtained two broad patterns of results. On the one hand, both robots were equally successful in capturing children’s attention, telling a story that children were subsequently able to narrate, and teaching the children new vocabulary items. On the other hand, as compared to the flat robot, the expressive robot provoked stronger emotional engagement in the story as it was being narrated, greater inclusion of the newly learned vocabulary into the retelling of the story and greater fidelity to the original story during the retelling. A plausible interpretation of these two patterns is that story narration *per se* was sufficient to capture children’s attention and sufficient to ensure encoding both of the story itself and of the new vocabulary. By contrast the mode in which the story was narrated—expressive or flat—impacted the extent to which the child eventually cast him or herself into the role enacted by the narrator. More specifically, it is plausible that children who were emotionally engaged by the expressive robot were more prone to re-enact the story-telling mode of the robot when it was their turn to tell the story to the puppet: they were more likely to reproduce some of the unfamiliar nouns that they had heard the robot use and more likely to mimic the specific phrases included in the robot’s narrative.

It is tempting to conclude that children identified more with the expressive robot and found it more appealing. It is important to emphasize, however, that no signs of that differentiation were apparent either in children’s explicit verbal judgments about the two robots or in the open-ended descriptions. In either case, children were quite positive about both of the robots. An important implication of these findings, therefore, is that children’s verbal ratings of the robots are not a completely accurate guide to the effectiveness of the robots as role models. Future research on social robots as companions and pedagogues should pay heed to such findings. More generally, the results indicate that it is important to assess the influence and impact of a robot via a multiplicity of measures rather than via questionnaires or self-report.

### Language Status

When analyzing children’s learning and performance based on their language status, we saw only modest differences. These differences—in which native English speakers and bilingual children correctly identified more target vocabulary words with both robots, and showed stronger phrase mirroring and use more target words than ELL students with the expressive robot—were not unexpected, given that bilingual and native English speaking children have greater familiarity with the language. Nevertheless, it is important to note that both native English speakers and ELL children who heard the story from the *Expressive* robot reused and retained more information from the robot’s story. Thus, despite the limitations listed in the following section, these results suggest that the storytelling activity was an effective intervention for all the native English speakers, the bilingual and the ELL children, leading to learning and engagement by all groups. This is an important finding given that ELL children arguably need the most additional support for their language development (Páez et al., 2007). Effective and engaging language learning interventions like this one that can benefit the entire classroom—native English speakers, bilingual, and ELL children alike—will be important educational tools in years to come.

### Limitations

We should note several limitations of this study. First, some potentially important individual differences among children, such as their learning ability, socio-economic status and sociability were not controlled. Second, although 45 children participated in the study ranging from 4 to 7 years, we did not have an equal number of children at each age. We also did not have an equal number of children with each language status. In future work, it will be important to assess a more homogenous sample, as well as the degree to which our results remain stable across these individual differences and across the preschool and elementary school years.

In addition, we did not have complete story retelling data for all children. As reported earlier, the audio quality of some of the recordings of children’s retells prevented analysis, and not all children performed delayed retellings. As a result of this and the aforementioned imbalances in age and language, the analyses we report here are under-powered. This is exploratory work, and the result should be interpreted in light of this fact. Future work should take greater effort to collect quality audio recordings and to see all children at the delayed test.

Finally, while the target vocabulary words used were uncommon, some children may still have known them—particularly older children, given the correlation between age and target words identified. The rarity of the words may have also increased their saliency, being a cue for children to pay attention to the words. Follow-up studies should either consider using nonce words or include a vocabulary pretest for the target words.

### Future Directions

The technology landscape continues to rapidly evolve from passively consumed content such as television and radio to interactive and social experiences enabled through digital technology and the internet. Each new technology transforms the ways we interact with one another, how we communicate and share, how we learn, tell stories and experience imaginary worlds.

Today, the linguistic and interpersonal environment of children is comprised of other people, yet children are increasingly growing up talking *with* AI-based technologies, too. Despite the proliferation of such technologies, very little is understood about children’s language acquisition in this emerging social-technological landscape. While it has been argued in the past that children cannot learn language from impersonal media because language acquisition is socially gated (e.g., Kuhl, 2007, 2011), the reality of social robots forces us to revisit our past assumptions. These assumptions need revisiting because numerous studies have now shown that children and adults interact with social robots as social others (Breazeal et al., 2008, 2016b; DeSteno et al., 2012). Social robots represent a new and provocative psychological category betwixt and between inanimate things and socially animate beings. They bridge the digital world of content and information to the physically co-present and interpersonal world of people. Because of this, we are likely to interact with social robots differently than prior technologies. As such, social robots open new opportunities for how educational content and experiences can be brought to the general public, just as their technological predecessors have.

Therefore, how *should* social robots be designed to best foster the learning, development, and benefit to children? This is very new territory, indeed. This work explores three key avenues, although there are many others to explore, and to explore deeply.

In the context of language learning for preschool age children, we begin by applying knowledge and taking inspiration from how children learn language through storytelling with a peer-like companion. Children learn quite a lot from interacting with and socially modeling the behavior and attitudes of their peers, and in prior work, we have seen behaviors suggesting that children also socially model or emulate the behavior of social robots. For instance, we have found that children become more emotively expressive when a robot is more expressive (Spaulding et al., 2016). We see this effect again in this work. We have also observed that when children play with a “curious” robot that exhibits pro-curious behaviors and attitudes, children express and engage in more curious behaviors (Gordon et al., 2015) and are more willing to teach new tasks to a robot peer (Park and Howard, 2015). In the present study, we found evidence of this social modeling effect in terms of children emulating the linguistic phrases and vocabulary a robot uses. This peer-learning dynamic is quite different from how children learn with other technologies.

Emotional expressivity is another characteristic that social robots bring to interaction. Understanding the impact of emotion and expressive behavior on learning with young children is an area worth further systematic investigation. It is generally accepted that telling a story more expressively will make it more engaging. Social robots enable us to study the impact of expressivity on children’s behavior and learning in a more systematic and carefully controlled way. Because of these attributes, social robots could serve as a compelling tool to gain insights into children’s social development and learning.

In this work, we observed a greater tendency for children to emulate a storytelling robot’s phrasing when the robot was more vocally expressive; children reproduced this pattern after a month-long delay. Further research is warranted to understand whether children are encoding the information differently when the delivery is more expressive, or whether they are simply more apt to emulate the robot when it is more expressive.

We see growing evidence that the more socially expressive and interactive a robot is, the more it “opens the spigot” to children’s social engagement and learning. This suggests a new paradigm for educational technology and how it promotes children’s learning and development. It is increasingly clear that it is not just the introduction of a social robot into an educational context that matters, *but how socially designed* the robot is that impacts children’s behavior and learning.

Finally, for social robots to have a large-scale impact in the educational realm, research should extend beyond the context of 1:1 interaction of a social robot with a child. We need to also understand how to design social robots to support and foster peer learning among groups of children; we need to understand how social robots can best support and include the participation of adults, such as teachers and parents, or facilitate classroom orchestration (e.g., Dillenbourg and Jermann, 2010); and we need to understand how to effectively integrate robots into the broader educational context of the classroom and continued learning at home. Much work remains to be done in order to understand how to best design social robots that can successfully engage and support learning over longitudinal time scales, where the opportunity to deeply attune to the individual child exists—not only in terms of curricular goals, but in order to foster positive attitudes toward learning and challenge, and to build trust and rapport as well. Finally, as research matures and social robots become an affordable mass consumer technology, there exists many opportunities for social robots to help support and augment learning experiences for children who are underserved, at-risk, or have other learning challenges.

## Author Contributions

The study was conceived and designed by JMKW, SJ, HWP, SR, PLH, DD and CLB. Data analysis was performed by JMKW, SJ, HWP, SR and AA. The article was drafted, written, revised and approved by JMKW, SJ, HWP, SR, AA, PLH, DD and CLB.

## Conflict of Interest Statement

The authors declare that the research was conducted in the absence of any commercial or financial relationships that could be construed as a potential conflict of interest.

## Footnotes

^1^http://affectiva.com/ (retrieved September 19, 2016).
